# The impact of knowledge about diabetes, resilience and depression on glycemic control: a cross-sectional study among adolescents and young adults with type 1 diabetes

**DOI:** 10.1186/1758-5996-5-55

**Published:** 2013-09-28

**Authors:** Fabio R Munhoz Santos, Viviane Bernardo, Monica A L Gabbay, Sergio A Dib, Daniel Sigulem

**Affiliations:** 1 Department of Health Informatics, Universidade Federal de São Paulo, Rua Botucatu, 862, São Paulo CEP:04023-062, SP, Brazil; 2 Endocrinology Division, Departament of Medicine, Universidade Federal de São Paulo, São Paulo, Brazil

**Keywords:** Glycemic control, Type 1 diabetes, Psychosocial factors, Depression, Resilience

## Abstract

**Background:**

The purpose of this study is to evaluate the relationship between glycemic control and the factors of knowledge about diabetes, resilience, depression and anxiety among Brazilian adolescents and young adults with type 1 diabetes.

**Methods:**

This cross-sectional study included 85 adolescents and young adults with type 1 diabetes, aged between 11–22 years, with an average age of 17.7 ± 3.72 years. Glycemic control degree was evaluated through HbA1c. To assess psychosocial factors, the following questionnaires were used: resilience (Resilience Scale, RS) and anxiety and depression (Hospital Anxiety and Depression Scale, HADS). The Diabetes Knowledge Assessment Scale (DKNA) was used to assess knowledge about diabetes.

**Results:**

Significant correlations were found between HbA1c and resilience, anxiety and depression. Multiple linear regression analysis revealed that the only variable which presented significant association with the value of HbA1c was depression.

**Conclusions:**

Depression has a significant association with higher HbA1c levels, as demonstrated in a regression analysis. The results suggest that depression, anxiety and resilience should be considered in the design of a multidisciplinary approach to type 1 diabetes, as these factors were significantly correlated with glycemic control. Glycemic control was not correlated with knowledge of diabetes, suggesting that theoretical or practical understanding of this disease is not by itself significantly associated with appropriate glycemic control (HbA1c ≤ 7.5%).

## Background

The importance of tight blood glucose control for the subsequent prevention of diabetes complications is well established: higher HbA1c levels in type 1 diabetes are associated with conditions such as retinopathy and nephropathy [1,2]. Despite the development of diabetes therapy during recent decades, the quality of diabetes care, in general requires improvement. Effective educational programs aimed at managing glycemic control need to address not only knowledge about the procedures, but also psychosocial factors [1].

In order to contribute to the development of educational activities for young people with type 1 diabetes, as well as promote knowledge about diabetes, this study assesses factors that have recognized scope and impact on glycemic control: on the one hand, depressive and anxiety symptoms; and on the other, resilience, which encompasses many protective factors such as social support, hope, self-efficacy, problem-focused strategies, etc.

The knowledge each patient has about diabetes is a basic element in educational programs for type 1 diabetes patients [3]. The patient is required to have basic knowledge about insulin, carbohydrate counting, diet, etc. Knowledge is one of many important variables involved in diabetes education. A study has demonstrated the positive impact that knowledge has on glycemic control [4]. However, others suggest that the level of knowledge about diabetes is not a predictor of good glycemic control and generally recommend the need for further research in this field [5,6].

The relationships between psychosocial factors and glycemic control in diabetic patients are well described in the literature. It is apparent that the chronicity of type 1 diabetes and the demands for management provide a fertile environment for adjustment problems. One of the most studied psychosocial factors in this area is depression. Depressive symptoms occur at higher levels in patients with diabetes than in the general population and are also associated with higher HbA1c levels [7-9]. Patients with diabetes who have clinical depression present higher rates of clinical complications, hospitalization and health expenditures [10]. In patients with type 1 diabetes, depression has a significant influence on treatment adherence and health outcomes [11].

Resilience is a psychosocial factor that has been gaining academic importance. It is a broad concept that encompasses an individual’s resources for adequately dealing with adversity and achieving positive results in such situations. Resilience involves optimism in the face of situations, strategies for problem-focused resolution, self-efficacy, and self-confidence [12]. In people with physical illnesses, resilience relates to factors such as self-care, adherence to treatment, quality of life as related to health, illness perception, pain perception and adherence to physical activity [13]. In a study of individuals with type 2 diabetes, DeNisco found that HbA1c levels and resilience scores had a significant negative correlation, suggesting that resilience may influence glycemic control [14]. In young people with diabetes, resilience is a protective factor, expressed to different degrees in each patient, when facing stressful situations [15].

The association of depression and diabetes mellitus is supported by numerous studies [6-10]. Some studies have evaluated the extent of resilience in diabetes patients [12-15]. However, we found no studies that simultaneously evaluated the relationship between glycemic control and knowledge about diabetes, depressive symptoms, anxiety and resilience in type 1 diabetes, particularly in young Brazilians. By better understanding the complex relationship between these factors and glycemic control, the present study will contribute to a greater understanding of the factors related to glycemic control in young people with type 1 diabetes and may also be useful for planning and implementing educational activities for such patients.

Thus, the present study aimed to (A) evaluate the correlation between glycemic control and knowledge about diabetes, resilience, anxiety, and depression in young Brazilians with type 1 diabetes and (B) verify the significant effect of each variable on glycemic control in a multiple linear regression analysis.

## Methods

### Research design

This was a cross-sectional study that included 90 adolescents and young adults with type 1 diabetes who were randomly invited to participate. These patients were being followed up at the outpatient public division of the Diabetes Center of the Federal University of São Paulo, São Paulo, Brazil. After Institutional Ethical Committee approval, interviews were conducted from May to September 2012. The inclusion criteria were as follows: diagnosis of type 1 diabetes, no mental disorders, absence of visual or hearing disabilities. The patients recruited had long-term type 1 diabetes diagnosed according to the American Diabetes Association criteria [16]. During the study, as in real life, they continued to adjust insulin doses based on glucose control, diet and exercise, according to the Brazilian Diabetes Society guidelines [17].

Of the invited patients, 85 (94.3%) agreed to participate in this study and 5 (5.7%) refused to participate, either because they did not want to participate or they lacked sufficient time to be interviewed. The age of the participants ranged from 11– 22 years old, with an average age of 1 7.7 ± 3.72 y.o (mean ± SD).

After signed consent was obtained, demographic data and the time of diagnosis were also collected. Informed consent was obtained from the patients over 18 years of age and from the family responsible, for patients under 18. On the same occasion, glycated hemoglobin (HbA1c) was evaluated and questionnaires were administered to assess the following variables: knowledge about diabetes, resilience, anxiety and depression.

All questionnaires were completed online at the Diabetes Center, and the data were stored in a database on the University’s server. The interviews took between 20 and 30 minutes and always occurred in the presence of a researcher and an assistant.

Table 1 describes the demographic profile of the sample. Among the 85 patients aged between 11–23 years old, 40 (53%) were women. Regarding their level of education, which relates to their instruction degree, 16 (19%) either were in progress or had completed the elementary school, 49 (57%) and 20 (24%) for secondary school and higher education, respectively. The duration of diabetes among patients was: 10 (11%) had been diagnosed in the last two years; 29 (34%) had been diagnosed between two and six years; and 46 (55%) had lived with a diagnosis of diabetes for over 6 years.

**Table 1:** Demographic profile of 85 type 1 diabetes patients

**Characteristic**	**n**	**%**
***Gender***		
Women	45	53%
***Age***		
11–18 years	41	48%
>18–23 years	44	52%
***Educational Level***		
Primary Education	16	19%
High School	49	57%
University	20	24%
***Duration of Diabetes***		
0–2 years	10	11%
>2–6 years	29	34%
>6 years	46	55%

### Methodology

#### Main variable

##### Glycemic control

The HbA1c was used as a parameter in order to evaluate the degree of glycemic control. Tests were performed within the 4 months preceding the interview. HbA1c was evaluated by high-performance liquid chromatography (HLPC), using a Tosoh G7 Auto HPLC. This method is certified by the National Glycohemoglobin Standardization Program - USA.

#### Secondary variables

##### Knowledge about diabetes

The Diabetes Knowledge Scale (DKN-A), developed by Beeney in 2000, was used to assess the level of knowledge about diabetes [18]; it was validated for Brazilian Portuguese by Torres et al. [19] and was also validated for use in young people and adolescents by Fitzgerald in 1998 [20].

##### Resilience

The resilience scale (RS) developed by Wagnild and Young in 1993 [21], and validated and adapted to Portuguese by Pesce in 2005 [22], was used. The RS questionnaire was also validated for use in young people and adolescents by Ahern in 2006 [23].

##### Anxiety and depression

Depressive symptoms were assessed using the Hospital Anxiety and Depression Scale (HADS), developed in 1983 by Zigmond and Snaith [24]. The scale consists of 14 questions; 7 items for depression (HADS-D) and 7 for anxiety (HADS-A). The scores for each subscale range from 0–21. This tool was validated for Portuguese by Gorenstein in 2000 [25], and was validated for use in young people and adolescents by White et al. in 1999 [26].

### Statistical analysis

Correlations between quantitative data were estimated using Pearson correlation. For ordinal data, the authors used the Spearman coefficient. A multiple linear regression model was prepared to simultaneously evaluate the effects of the factors (knowledge about diabetes, resilience, depression and anxiety) on HbA1c. The level of significance was set at *p* ≤ 0.05. Data were analyzed using the SPSS software package version 18.0.

## Results

The HbA1c level in subjects in the present study was 9.3 ± 2. 3% (mean ± sd), ranging from 5.8 to 16.1%. Of the subjects, 65 (76.5%) had HbA1c levels >7.5%, and 20 (23.5%) ≤ 7.5%.

### Relationships between HbA1c and the variables evaluated

Table 2 presents the correlations between the study variables and HbA1c levels.

**Table 2:** Correlation between HbA1c and evaluated factors (knowledge, resilience depression and anxiety) in the patients sample (n = 85)

	**Knowledge**	**Resilience**	**Anxiety**	**Depression**
	**(DKNA)**	**(RS)**	**(HADS-A)**	**(HADS-D)**
HbA1c	−0.06	−0.22	0.25	0.33
	(0.600)	(0.048)*	(0.022)*	(0.002)*
Knowledge (DKNA)	-	0.20	0.05	0.02
		(0.065)	(0.642)	(0.876)
Resilience (RS)	-	-	−0.36	−0.32
			(<0.001)**	(0.003)*
Anxiety (HADS-A)	-	-	-	0.51
				(<0.001)**

Data represent Pearson correlation coefficients and their statistical significance (in parentheses). *HbA1c*: glycated hemoglobin, *DKNA*: Diabetes Knowledge Assessment Scale, *HADS-A*: HADS anxiety score, *HADS-D*: HADS depression score. (* p < 0.05 ; ** p < 0.001).

### Impact of each variable on glycemic control

Table 3 shows the results of the multiple linear regression analysis, assessing the impact of each variable on HbA1c.

**Table 3:** Impact of factors (knowledge, resilience depression and anxiety) on HbA1c levels in a multiple linear regression model in the patients sample (n = 85)

**Factor**	***B***	**95% CI**	***p***	***r***_**(partial)**_
Constant	9.97	-	-	
Knowledge (DKNA)	−0.05	−0.24 to 0.14	0.603	−0.06
Resilience (RS)	−0.04	−0.12 to 0.04	0.306	−0.12
Anxiety (HADS-A)	0.07	−0.10 to 0.24	0.418	0.09
Depression (HADS-D)	0.20	0.02 to 0.37	0.031*	0.24

B is the estimate of how much a one-unit increase in a factor is associated with the variation in HbA1c levels. The partial “*r*” represents the correlation of the factor with HbA1c after considering the effect of the other terms in the model. *HbA1c*: glycated hemoglobin, *DKNA*: Diabetes Knowledge Assessment Scale, *HADS-A*: HADS anxiety score, *HADS-D*: HADS depression score. (* p < 0.05).

## Discussion

The results of the present study among Brazilian adolescents and young adults with type 1 diabetes show that 76.5% of the subjects had an HbA1c level above 7.5%. In this sample, the mean HbA1c was 9.3 ± 2.3% (mean ± sd). The goal of treatment for young people and adolescents with type 1 diabetes is an HbA1c value of 7.5% or less [2]. A high percentage of this study’s population had suboptimal glycemic control (HbA1c ≥ 7.5%), and thus was within the risk range for the development of diabetic complications [2]. This finding confirms the need for constant efforts to understand the factors that have an impact on glycemic control, aiming at promoting educational activities for this population.

Among the variables assessed, only depressive symptoms were significantly associated with high HbA1c in a regression analysis. This result is consistent with the literature and corroborates studies which show a link between depression, higher HbA1c levels, hospitalization and complications [7-10,27]. This is probably because depression carries low self-esteem and neglect of self-care behaviors. We can infer that a patient with depressive symptoms will not take care of glycemic monitoring, and may become careless with diet and physical activities.

The anxiety scores in the study subjects were significantly correlated with HbA1c, but were not significantly associated in a multiple regression analysis. Shabam et al. report a different outcome in type 1 diabetes adults via a multiple regression analysis, showing anxiety as a predictor of high HbA1c; they also used the HADS [27]. Anxiety symptoms are more common in patients with diabetes than in the general population [28]. The results of these studies converge on the same implication: anxiety is a significant factor that must be taken into account and addressed in glycemic control programs.

The results of the present study indicate that resilience is also significantly correlated with HbA1c. This result is concordant with the preliminary study of Jaser, which shows that good glycemic control (HbA1c ≤ 7.5%) is associated with resilience and the use of appropriate coping strategies (e.g., problem solving, emotional expression, acceptance, and social support) [29]. In a literature review, Bradshaw states that resilience can be increased in patients with diabetes; although these resilience trainings do not demonstrate a direct impact on glycemic control, they result in improvements in self-care behaviors [30]. It can be inferred from these results that training in resilience should be incorporated into programs for this population, covering topics such as: optimal sleep, physical activity, appropriate food choices, blood glucose monitoring, compliance with medication, self-efficacy and social support.

In this study, the level of knowledge about diabetes was not significantly correlated with HbA1c. This indicates that both well- and poorly-controlled subjects exhibit similar levels of basic knowledge about diabetes. Previous studies also found no correlation between knowledge about diabetes and glycemic control [5,6,31].

Although knowledge is not significantly associated with glycemic control, it is a prerequisite for a patient to perform appropriate self-care [4]. Knowledge education per se may not, however, be the major operative factor in improving control of diabetes. Strategies to provide information must be combined with other behavioral strategies to motivate and help patients effectively manage their diabetes.

### Study limitations

There are a few important limitations to this study. The first limitation is the inability to assess causal factors due to the study’s cross-sectional design. Another limitation is that variables known to have an effect on glycemic control were not examined in the current research, for example, social networks, self-efficacy, self-esteem, and family environment.

Future longitudinal studies are needed to explore the causal relationships of these factors, as well as studies assessing the effectiveness of interventions that contribute to better glycemic control in this population.

## Conclusions

The high percentage of patients with higher HbA1c levels (76.5% with HbA1c > 7.5%) found in the present study implies that there is a need for effective multidisciplinary actions to improve the glycemic control of these adolescents and young adults with type 1 diabetes. It is important that diabetes care groups develop and evaluate specific programs for this population. Glycemic control was not correlated with knowledge of diabetes, suggesting that only theoretical or practical understanding about this disease is not associated with good glycemic control. It can be concluded that information by itself is not significantly associated with good control.

As this study has shown, depression has a significant association with higher HbA1c levels and anxiety and resilience are significantly correlated with glycemic control. These factors should be addressed in educational programs aimed at improving glycemic control in adolescents and young adults with type 1 diabetes.

## Abbreviations

HbA1c: Glycated hemoglobin; HLPC: High-performance liquid chromatography; HADS: Hospital anxiety and depression scale; DKN-A: Diabetes knowledge scale; RS: Resilience scale; HHI: Herth hope index.

## Competing interests

The authors declare that they have no competing interests.

## Authors’ contributions

FRMS wrote the paper, collected data and performed statistical analyses; SAD and MALG assisted in patient recruitment; DS, VB and SAD assisted in conception of the research hypothesis and reviewed the manuscript. All authors read and approved the final manuscript.
